# SEOM–GEICO clinical guideline on epithelial ovarian cancer (2023)

**DOI:** 10.1007/s12094-024-03531-3

**Published:** 2024-07-15

**Authors:** Jose Alejandro Perez-Fidalgo, Fernando Gálvez-Montosa, Eva María Guerra, Ainhoa Madariaga, Aranzazu Manzano, Cristina Martin-Lorente, Maria Jesús Rubio-Pérez, Jesus Alarcón, María Pilar Barretina-Ginesta, Lydia Gaba

**Affiliations:** 1 Hospital Clínico Universitario, Biomedical Research Institute INCLIVA, University of Valencia, Valencia, Spain; 2 Hospital Universitario de Jaén, Jaén, Spain; 3 Hospital Universitario Ramon y Cajal, Madrid, Spain; 4 Department of Medical Oncology, 12 de Octubre University Hospital, Madrid, Spain; 5 Hospital Clínico San Carlos, Madrid, Spain; 6 Hospital de la Santa Creu i Sant Pau, Barcelona, Spain; 7 Hospital Universitario Reina Sofía, Universidad de Córdoba (UCO), Córdoba, Spain; 8 Hospital Universitari Son Espases, Mallorca, Spain; 9 Institut Català d’Oncologia, Medical Oncology Department, Precision Oncology Group, Institut d’Investigació Biomèdica de Girona (IDIBGI), Medical Sciences Department, Universitat de Girona, Girona, Spain; 10 Medical Oncology Department, Hospital Clinic, Translational Genomics and Targeted Therapies in Solid Tumors, IDIBAPS, Department of Medicine, University of Barcelona, Barcelona, Spain

**Keywords:** Ovarian cancer, Guideline, Diagnosis, Treatment

## Abstract

In recent years, the incorporation of new strategies to the therapeutic armamentarium has completely changed the outcomes of epithelial ovarian cancer (EOC). The identification of new predictive and prognostic biomarkers has also enabled the selection of those patients more likely to respond to targeted agents. Nevertheless, EOC is still a highly lethal disease and resistance to many of these new agents is common. The objective of this guideline is to summarize the most relevant strategies to manage EOC, to help the clinician throughout the challenging diagnostic and therapeutic processes and to provide evidence-based recommendations.

## Introduction

EOC represents a heterogeneous disease with clinically, pathologically, and clinically different tumours. Histological subtype, stage at diagnosis, molecular biomarkers, and access to appropriate surgery and systemic therapy in specialized centres are crucial factors that will impact outcomes. Cytoreductive surgery with no macroscopic residual disease and combination of platinum–taxane chemotherapy (ChT) remain the mainstay of therapy. Maintenance treatment with antiangiogenics and/or poly (adenosine diphosphate-ribose) polymerase (PARP) inhibitors has proven to exert an important impact on clinical outcomes [1].

The incorporation of molecular biology to identify predictive biomarkers into standard practice will enable selection of those patients who would benefit most from targeted agents. Therapeutic options in the setting of recurrence remain limited, which highlights a substantial unmet need [1].

This SEOM–GEICO guideline provides updated evidence-based recommendations for the current treatment of EOC, primary peritoneal, and fallopian tube cancer, globally considered as EOC throughout this guideline.

## Methodology

This guideline is based on a systematic review of relevant published studies and with the consensus of ten oncologists who are experts in the treatment of these neoplasms from GEICO (Grupo Español de Investigación en Cáncer Ginecológico) and SEOM (Sociedad Española de Oncología Médica), as well as an external review panel of two experts designated by SEOM. The Infectious Diseases Society of America–US Public Health Service Grading System for Ranking Recommendations in Clinical Guidelines has been used to assign levels of evidence and grades of recommendation.

Final recommendations on each chapter are based solely on those drugs approved by the EMA and/or FDA.

### Incidence and epidemiology

EOC is the second most deadly gynaecological cancer worldwide and the first in developed countries, responsible for some 200,000 deaths annually. In Spain, 1979 women died of OC in 2021, and approximately 3584 new cases were diagnosed during 2023 [2]. Median age at the time of diagnosis is approximately 63 years.

Nulliparity, obesity, and treatment with oestrogen therapy are known risk factors for EOC. Genital use of talc powder has been suggested as a potential risk factor. In contrast, higher parity, oral contraceptive use, and breastfeeding have a protective role. Tobacco have been studied without conclusive results [3].

Moreover, high grade serous (HGS) carcinomas are strongly associated with family history and hereditary syndromes. Mutations in the *BRCA1/2* genes, which are detected in 10–15% of patients with OC and cause Hereditary Breast and Ovarian Cancer syndrome, are associated with a 15–65% risk of EOC, especially HGS histology. Mutations in the *MLH1*, *MSH2*, *MSH6*, and *PMS2* genes (diagnostic of Lynch syndrome), also correlate with a 12% risk of developing OC, mainly endometrioid or clear cell. Mutations in the *ATM*, *BRIP1*, *PALB2*, *RAD51C* and *RAD51D* genes are associated with a moderate risk of developing OC [4].

### Diagnosis and staging

More than two-thirds of all cases are diagnosed at an advanced stage, given that the symptoms of early stage EOC are not specific. Two large prospective trials, the UKTCTOCS and PLCO trials that enrolled 202,562 and 78,216 women, respectively, found that a screening program for OC had no clear impact on mortality [5].

Common symptoms of EOC include abdominal/pelvic pain, constipation, urinary frequency, abdominal distension, shortness of breath, and fatigue. The initial evaluation includes a physical examination, laboratory testing including CA 125, and pelvic ultrasound. Elevated HE4 levels identify malignancy with a sensitivity similar to that of CA 125, albeit with greater specificity. Algorithms such as the International Ovarian Tumour Analysis (IOTA) Simple Rules risk model or IOTA Assessment of Different NEoplasias in the adneXa (ADNEX) model can be useful in distinguishing benign from malignant pelvic tumours.

Computed tomography (CT) imaging of the thorax, abdomen, and pelvis defines the extent of the disease and informs treatment planning. If available, magnetic resonance imaging (MRI) and positron emission tomography (PET)–CT can enhance assessment accuracy in advanced disease. Initial laparoscopy is a mainstay for histopathological diagnosis and to evaluate the likelihood of complete cytoreduction [6]. Peritoneal extension scores, such as the peritoneal cancer index or the Fagotti score could be useful in this context. An adequate amount of tissue is essential to establish a pathological diagnosis and analyse biomarkers to guide the treatment plan. Cytological testing of ascites and pleural fluid, if present, is required to complete staging. FIGO 2014 [7] is the staging system currently recommended (Table 1).

**Table 1:** 2014 FIGO staging system for ovarian, fallopian tube, and peritoneal cancer

Stage I	Tumour confined to ovaries or fallopian tube(s)
IA	Tumour limited to one ovary (capsule intact) or fallopian tube; no tumour on ovarian or fallopian tube surface; no malignant cells in the ascites or peritoneal washings
IB	Tumour limited to both ovaries (capsules intact) or fallopian tubes; no tumour on ovarian or fallopian tube surface; no malignant cells in the ascites or peritoneal washings
IC1	Tumour limited to one or both ovaries or fallopian tubes, with surgical spill
IC2	Tumour limited to one or both ovaries or fallopian tubes, with capsule ruptured before surgery or tumour on ovarian or fallopian tube surface
IC3	Tumour limited to one or both ovaries or fallopian tubes, with malignant cells in the ascites or peritoneal washings
Stage II	Tumour involves one or both ovaries or fallopian tubes with pelvic extension or primary peritoneal cancer
IIA	Extension and/or implants on uterus and/or fallopian tubes and/or ovaries
IIB	Extension to other pelvic intraperitoneal tissues
Stage III	Tumour involves one or both ovaries or fallopian tubes, or primary peritoneal cancer, with cytologically or histologically confirmed spread to the peritoneum outside the pelvis and/or metastasis to the retroperitoneal lymph nodes
IIIA1	Positive retroperitoneal lymph nodes only (cytologically or histologically proven)
IIIA1 (i)	Metastasis up to 10 mm
IIIA1 (ii)	Metastasis more than 10 mm
IIIA2	Microscopic extrapelvic (above the pelvic brim) peritoneal involvement with or without positive retroperitoneal lymph nodes
IIIB	Macroscopic peritoneal metastasis beyond the pelvis up to 2 cm in greatest dimension, with or without metastasis to the retroperitoneal lymph nodes
IIIC	Macroscopic peritoneal metastasis beyond the pelvis more than 2 cm in greatest dimension, with or without metastasis to the retroperitoneal lymph nodes (includes extension of tumour to capsule of liver and spleen without parenchymal involvement of either organ)
Stage IV	Distant metastasis excluding peritoneal metastases
IVA	Pleural effusion with positive cytology
IVB	Parenchymal metastases and metastases to extra-abdominal organs (including inguinal lymph nodes and lymph nodes outside of the abdominal cavity)

### Recommendations

- Screening for EOC in average risk women is not recommended (I, E).
- The initial evaluation of a patient with suspicion of EOC should include physical examination, laboratory testing with CA-125, and pelvic ultrasound [II, A].
- CT of the thorax, abdomen, and pelvis is recommended in the diagnostic workup to assess the extent of the disease (II, A).
- Laparoscopic surgery is recommended in advanced EOC to evaluate cytoreduction, obtain material for pathologic diagnosis and predictive biomarkers (II, B).

### Pathology and molecular biology

EOC is the most common ovarian cancer histology (~ 90) and can be classified into five main subtypes according to the 2020 World Health Organization (WHO) classification: high-grade serous (HGS, 70%), low grade serous (LGS, 10%), endometrioid carcinoma (EC, 10%), clear cell carcinoma (CCC, 5%), and mucinous carcinoma (MC, 3%). Other less frequent histologies (2–3%) include undifferentiated carcinoma, malignant Brenner tumour, mesonephric-like carcinoma, mixed carcinomas, or carcinosarcoma [8]. Diagnosis should be made by an expert gynaecological pathologist by immunohistochemistry (IHC) to avoid misdiagnoses. Each EOC subtype depends on a different molecular background (Table 2) that can address different pathogenesis, clinical features, response to treatments, and prognosis [9].

**Table 2:** IHC characteristics and molecular background of EOC

	PAX8	WT1	p53 abnormal*	Napsin A	Hormonal receptors	Molecular background**
HGS	Strong +	+	Yes	−/+	−/+	TP53, BRCA1/2, HR genes, RB1, NOTCH and PI3K pathway
LGS	Strong +	+	No	−	+ +	BRAF, KRAS, MAPK pathways
EC	Moderate +	Variable	Variable	−/+	−/+	PTEN, PI3K pathway, KRAS, Wnt/beta-catenin pathway
CCC	Strong +	−	Variable	+	−	SWI/SNF genes (i.e. ARID1A), PTEN, PI3K pathway, MMR pathway
MC	−/+	–	No	–	Variable	KRAS, ERBB2 amplification

*HGS* high-grade serous, *LGS* low-grade serous, *EC* endometrioid carcinoma, *CCC* clear cell carcinoma, *MC* mucinous carcinoma, *MMR* mismatch repair

*Abnormal p53: nuclear overexpression or complete absence of expression; **mutations and deregulated pathways

*BRCA1/2* mutational status should be determined at primary diagnosis in every non-mucinous EOC regardless of age at diagnosis or family history of cancer. Although less common, Lynch syndrome can be associated with mucinous and non-mucinous EOC. Mismatch repair (*MMR*) genes testing is highly recommended depending on the family history of cancer. More extensive germline next generation sequencing (NGS) panels should be offered, depending on clinical suspicion [4].

Homologous recombination deficiency (HRD) provides prognostic information and can be used as a predictive biomarker of the magnitude of response to PARP inhibitors (PARPi). Commercially available NGS tests detect genetic scars and somatic *BRCA1/2* mutations as subrogates of HRD and are the most common way to test homologous recombination status in clinics. Different cutoffs can be observed depending on the test. HRD status should be assessed at diagnosis in at least every high-grade EOC patient (i.e., serous and endometroid subtype) [10].

### Recommendations

- Initial IHC-based diagnosis should be made by a gynaecological pathology expert [IV, A].
- All patients with non-mucinous EOC should be tested for somatic and/or germline *BRCA1/2* mutation at primary diagnosis to discard a hereditary syndrome, to obtain prognostic information, and to select a biomarker of response to PARPi [I, A].
- Determination of HRD with a clinically validated test is strongly recommended at initial diagnosis in every high-grade serous or endometrioid EOC to provide prognostic and predictive biomarker information [I, A].MMR testing and/or more extensive germline NGS panels are recommended depending on clinical suspicion and family history of cancer [II, A].

### Management of early stage disease (FIGO stages I–II)

#### Surgery

**Fig. 1:**
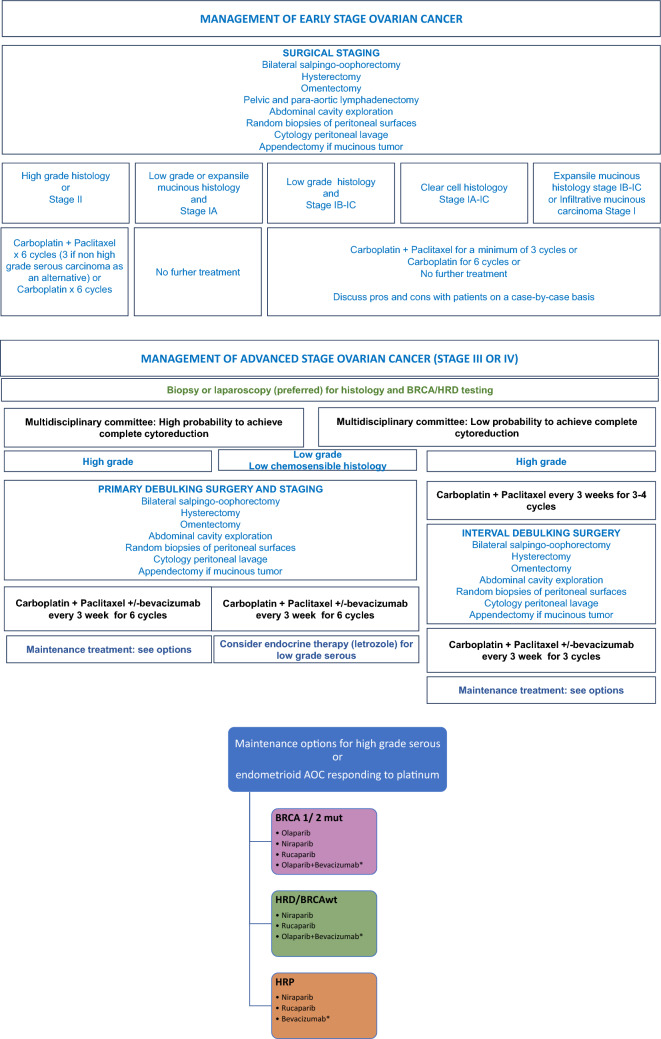
Management of early and advanced stage EOC

Treatment of early EOC requires surgery including hysterectomy, bilateral salpingo-oophorectomy, systematic pelvic and para-aortic lymphadenectomy, omentectomy, appendectomy in MC, random peritoneal biopsies of all surfaces, peritoneal washings with cytological examination, and complete exploration of the peritoneal cavity (Fig. 1).

In FIGO stage I, low-grade endometroid and expansile mucinous due to a rate of lymph node involvement < 1% systematic pelvic and para-aortic lymphadenectomy is not recommended.

The standard approach is by supra- and infra-umbilical median laparotomy. The laparoscopic approach is being investigated, but we currently lack prospective data demonstrating its equivalence to open surgery [1]. The aim of the surgery is to remove the entire tumour and stage the disease so as to establish the indication for subsequent adjuvant treatment.

Fertility-preserving surgery could be considered in stage IA or IC disease with unilateral ovarian involvement and low histologic grade [11].

### Chemotherapy

Adjuvant platinum-based ChT after complete surgery will be offered to all patients diagnosed with a high-grade tumour or stage II disease, based on long-term follow-up results from the ICON1-ACTION studies.

Adjuvant ChT is not recommended in completely staged patients with LGS stage IA, low-grade EC stage IA, or expansile MC stage IA–IB. The benefit of adjuvant ChT is uncertain and could be regarded as optional in LGS stage IB–IC, CCC stage IA–IC1, low-grade EC stage IB–IC, expansile MC stage IC, and infiltrative MC stage IA [1].

Standard adjuvant treatment consists of six cycles of platinum-based ChT. However, there is no consensus regarding treatment duration or optimal schedule. The GOG 157 study compared three vs. six cycles of carboplatin and paclitaxel. The study revealed no difference in recurrence rate, although a subsequent analysis did demonstrate a benefit in HGS in favour of the longer treatment; nevertheless, this group represented only 23% of the study population [12].

Although the most commonly used schedule is the platinum and taxane combination, there is no evidence that adding paclitaxel is of benefit in adjuvant treatment of early ovarian cancer. Thus, carboplatin monotherapy may be an appropriate option in this setting. The magnitude of benefit as well as short- and long-term toxicity should be discussed with the patient prior to deciding on the adjuvant scheme to be used [13].

### Recommendations

- Surgery with complete tumour resection and complete surgical staging is the mainstay for both treatment and to establish the extent of the disease that informs the subsequent indication for systemic treatment (I, A).
- Adjuvant platinum-based ChT is recommended in all cases of high grade or stage II (IA). The most widely used option is the combination of carboplatin and paclitaxel, although carboplatin monotherapy may be acceptable (II, A).
- Treatment duration should be at least three cycles for all subtypes, albeit six cycles are recommended in HGS (II, A).

### Management of advanced-stage disease (AOC) (FIGO stages III–IV)

#### Cytoreductive surgery

The recommended strategy in individuals with stage III–IV disease is primary debulking surgery (PDS), followed by systemic treatment [1].

Cytoreduction surgery in advanced EOC (FIGO stages III–IV) has a proven therapeutic purpose. Its goal must be complete excision (R0) of any visible tumour without leaving macroscopic residual disease, given that post-surgical volume will impact the risk of recurrence and patient survival [14].

In light of the results of the LION and the CARACO trials, lymphadenectomy in primary fully resected AOC with clinically negative lymph nodes is no longer recommended nor in upfront nor in interval cytoreductive surgery [15, 16].

Surgical expertise and specialist training are known to result in better rates of complete cytoreduction. Hence, subjects with advanced disease are advised to undergo surgery in specialized centres with suitable infrastructure and trained teams [17].

### Chemotherapy

Conventional treatment is based on a combination of carboplatin (AUC 5–6) and paclitaxel (175 mg/m^2^) every 3 weeks for 6 cycles**.** Schedules with weekly ChT without bevacizumab improve neither progression-free survival (PFS) nor overall survival (OS) in patient populations from Western countries [19]. Weekly carboplatin–paclitaxel improved QoL vs. 3-weekly suggesting a role in elder patients. However in a study in vulnerable elderly patients single-agent carboplatin or weekly ChT might have worse outcomes; consequently, the 3-week regimen remains the standard for all cases of AOC including the elderly [20].

Prophylaxis of venous thromboembolism in advanced EOC patients should be discussed with each patient receiving systemic therapy considering their specific risk factors [18].

### Neoadjuvant chemotherapy

The EORTC55971 trial and CHORUS trial found similar PFS and OS rates for patients with stage IIIC or IV disease receiving neoadjuvant chemotherapy (NACT) and interval debulking surgery (IDS) compared with PDS. Despite these non-inferiority results, the aforementioned trials have been criticized for their short median OS, mean operative time, and low optimal cytoreduction rates.

Due to the limitations of these trials, it has yet to be determined whether NACT and IDS might be an option for people for whom complete resection at PDS seems feasible. Therefore, both approaches (PDS or NACT followed by IDS) may be deemed valid, although PDS is still the preferred primary treatment option when complete cytoreduction is feasible and patient is operable [1].

### Intraperitoneal chemotherapy

Despite the fact that three large, randomized studies (GOG 104, GOG 114, and GOG 172) and one meta-analysis have found clinically significant improvements in PFS and OS with intraperitoneal (IP) ChT [21], the results of the GOG 252 trial revealed no benefit of IP therapy when bevacizumab was incorporated in all arms. Therefore, IP chemotherapy is not considered a standard of care [1].

A randomized phase III trial evaluating hyperthermic intraperitoneal chemotherapy (HIPEC) after IDS evidenced better PFS and OS for the HIPEC arm. Nevertheless, this trial received notable methodological criticisms, in particular, the lack of stratification for known prognostic molecular factors. Therefore, HIPEC cannot be regarded as a standard treatment and should not be offered outside of the context of clinical trials [1].

### Maintenance treatment with bevacizumab

Two large, randomized studies (GOG 218 and ICON 7) have reported that bevacizumab (15 mg/kg or 7.5 mg/kg every 3 weeks) added to adjuvant ChT after PDS and followed by maintenance therapy with bevacizumab for a maximum of 15 months improves PFS compared to standard adjuvant ChT alone. Post-hoc subgroup analyses indicated statistically significant OS benefit only in patients with stage IV disease in GOG 218 [22] and patients at high risk of progression (defined as FIGO stage III with > 1 cm residual disease following PDS or stage IV) in the ICON7 trial [23].

In the ENGOT Ov-15 study, prolonging the duration of bevacizumab administration (30 vs. 15 months) failed to improve PFS [24].

Two small, prospective trials revealed that bevacizumab added to platinum-containing ChT in the neoadjuvant setting was safe, although it has no impact on complete resection rate or PFS [1].

### Maintenance treatment with PARP inhibitors

Several phase III, randomized clinical trials (SOLO 1, PRIMA, PAOLA, PRIME, and ATHENA–MONO) have demonstrated that maintenance therapy with PARPi (olaparib, niraparib, olaparib plus bevacizumab, and rucaparib) after response to front-line platinum-containing regimens significantly increased median PFS in HGSOC [25–29].

All trials have manifested a remarkable, unprecedented benefit inBRCA1/2 mutated individuals. Moreover, olaparib–bevacizumab, niraparib, and rucaparib also displayed a significant benefit in the HRD population. Finally, niraparib and rucaparib exhibited a benefit in the HR proficient (HRP) and HRD-unknown subgroups, albeit of lesser magnitude.

The benefit observed with PARPi has been sustained throughout follow-up as demonstrated by their impact on PFS2, as well as by the results of long-term overall survival in the SOLO-1 and PAOLA-1 trials, with survival rates of 67% at 7 years and 65.5% at 5 years in the experimental arm vs. 46.5% and 48.4% in the control arm, respectively. The benefit was observed despite the fact that 40% of the subjects in the control group received subsequent PARP therapy [30, 31]. ATHENA-MONO trial also showed that rucaparib imrpoved PFS in all populations regardless of BRCA or HRD status having received a recent EMA approval for the indication of maintenance in first line.

Based on these results, olaparib (with or without bevacizumab), rucaparib or niraparib after partial or complete response to first-line, platinum-based ChT are highly effective in BRCA1/2-mutated patients and are strongly recommended.

According to the outcomes of the PAOLA-1 and PRIMA and ATHENA trials, niraparib, rucaparib or olaparib–bevacizumab are also highly recommended for women with HRD tumours.

In the HRP subgroup, maintenance with niraparib, or rucaparib can also be considered, although bevacizumab remains a reasonable alternative.

The choice of maintenance treatment should be based on: (1) molecular biomarkers (*BRCA1/2* and HRD status), (2) disease-related factors (stage at diagnosis, post-surgical residual disease, response to ChT, chemotherapy response score (CRS), CA-125 ELIMination Rate Constant K (KELIM)), and (3) patient characteristics (comorbidities, concomitant medication).

### Advanced, non-high grade serous ovarian cancer

Paclitaxel–carboplatin ± bevacizumab is the standard systemic ChT used in non-HGSC**.** Multiple retrospective studies, however, demonstrated lower response rates in these histologic subtypes compared with HGSC [1]. Due to the lower chemosensitivity, PDS is strongly recommended in uncommon histologies. Most LGSCs have high expression of oestrogen (ER) and progesterone receptors (PgR). Retrospective studies suggest a possible therapeutic value of hormone therapy in the maintenance of newly diagnosed advanced LGSC [32][IV, B].

Uncommon non-HGS histologies are an unmet need and inclusion of these patients in clinical trials is fervently encouraged.

### Recommendations

- Cytoreductive surgery aimed at achieving complete cytoreduction (absence of all visible residual disease) is the backbone of treatment for advanced EOC and should be performed in specialised centres [II, A].
- Lymphadenectomy in primary, completely debulked AOC with clinically negative lymph nodes is not recommended [I, A].
- Standard treatment is based on a combination of carboplatin (AUC 5–6) and paclitaxel (175 mg/m^2^) every 3 weeks for 6 cycles [I, A].
- If complete cytoreductive surgery is not feasible, NACT for three cycles followed by ICS and three cycles of ChT is recommended [I, A].
- IP ChT and HIPEC are not considered standard of care [I, D].
- The addition of bevacizumab to ChT should be contemplated, especially in patients with stage III and residual disease or stage IV [I, A].
- Post-ChT maintenance treatment is recommended after in HG-EOC. PARPi are recommended only after partial or complete response or no evidence of disease after first-line platinum-based ChT:
  o In BRCA1/2-mutated subjects: single agent with olaparib, rucaparib or niraparib or combination with olaparib plus bevacizumab  [I, A]
  o HRD tumours: niraparib, rucaparib or olaparib–bevacizumab [I, A]
  o HRP tumours: Niraparib, rucaparib [I, B] or bevacizumab [I, A]
- Paclitaxel–carboplatin ± bevacizumab is the standard systemic ChT used in non-HGSC [I, B].
- In newly diagnosed advanced LGSC maintenance treatment after Cht with hormone therapy can be considered [IV, B].

### Management of recurrent disease

Approximately 85% of all individuals diagnosed with advanced EOC experience disease recurrence within 10 years. Choosing the optimal strategy for recurrent ovarian cancer (ROC) demands that several critical factors be evaluated. Treatment-free interval (TFI) following last-platinum (TFIp) therapy remains a pivotal prognostic factor. Nevertheless, TFI should not be the sole consideration when making clinical decisions, since other clinical and molecular characteristics can impact treatment response and must be pondered [33].

Factors to consider in treatment assessment are histological subtype, BRCA1/2 status, extension of the disease and symptoms, feasibility and outcome of a potential second surgery, prior treatment and response, TFI from the last treatment, residual toxicity from previous therapeutic interventions, patient condition and comorbidities, and patient preferences [33].

### Surgery for relapse of ovarian cancer

The role of secondary cytoreduction (SC) in patients with first relapse more than 6 month TFIp has been examined in the DESKTOP III trial [34]. SC followed by ChT in cases selected on the basis of a favourable AGO score defined as a good performance status (ECOG 0), no residual disease after PDS, and the absence of ascites (< 500 ml) demonstrated an improvement in PFS and OS compared to ChT alone. The SOC-1 trial also assessed the role of SC in patients selected by an iModel algorithm. This trial evidenced a benefit in PFS from surgery, but OS data are still immature [35].

In contrast, the GOG-0213 trial failed to prove superiority of SC. The absence of well-defined selection criteria for surgical intervention questions the negative outcomes of the study [36].

Patient selection appears to be crucial to identify those who will benefit from this strategy.

### Systemic treatment when platinum is the best option

#### Chemotherapy

Platinum is considered the best treatment option for individuals who do not exhibit progression during previous treatment with platinum, do not present early symptomatic relapse, or who have no contraindication to platinum. Carboplatin doublets have an increased PFS and OS compared to carboplatin monotherapy [1]. Likewise, association with paclitaxel, gemcitabine, or pegylated liposomal doxorubicin (PLD) have demonstrated similar efficacy, with the choice of companion agent being based on the patient's previous toxicity profile and preferences.

For individuals with previous hypersensitivity to platinum, there are validated desensitization protocols that can be used under supervision if platinum is deemed the best option [37]. The INOVATYON study compared a non-platinum doublet (trabectedin–PLD) to a platinum doublet (carboplatin–PLD) [38] in patients with a TFIp of 6–12 months and failed to prove an increase in OS. Therefore, a platinum doublet is the preferred option in the first relapse.

### Maintenance treatment

*Antiangiogenic treatment* Bevacizumab, in combination with a platinum doublet with gemcitabine or paclitaxel and then as a maintenance treatment, has demonstrated increased percentages of objective responses and PFS compared to ChT and placebo. Carboplatin–PLD–bevacizumab increased PFS and OS vs. carboplatin–gemcitabine–bevacizumab, making it the preferred option for patients who have not received prior antiangiogenic treatment [39].

Although not authorized in Europe, in individuals with TFIp > 6 months who have previously received bevacizumab, retreatment with bevacizumab increased PFS with respect to ChT alone [40].

*PARP inhibitors* Three PARPi (olaparib, niraparib, and rucaparib) have been approved as maintenance therapy for subjects with ROC and response to platinum. Olaparib significantly increased PFS vs. placebo in those with *BRCA1/2* mutations in the SOLO2 study [41]. In the NOVA study [42], niraparib increased PFS in the *BRCA1/2* mutated population, *BRCA* non-mutated patients with HRD, as well as in the overall non-*BRCA* cohort. In the ARIEL3 study [43], rucaparib demonstrated better PFS in *BRCA1/2* mutated cases, the HRD population, and in the intention-to-treat population.

The recent communication of results of longer OS follow-up has generated controversy as to whether maintenance with PARPi could be harmful in the long-term in non-germline *BRCA* (g*BRCA*) mutation carriers, and affect response to subsequent retreatment with platinum, thereby generating resistance [44]. Nonetheless, OS was not a primary objective in these studies nor were they powered to do so. Consequently, the European Medicines Agency (EMA) continues to endorse niraparib and rucaparib as maintenance treatments in non-g*BRCA* mutation carriers. Nevertheless, the risks and benefits should be discussed with patients.

Retreatment with iPARP has been explored in the OREO study [45] which found a modest PFS benefit in selected patients; albeit it is not currently approved.

### Systemic treatment when platinum is not the best option

In patients with ROC that develop progressive disease while on platinum-based ChT or shortly thereafter, platinum rechallenge may not be an option. Patients with a short TFIp (< 6 months) have often been treated with multiple prior lines of therapy and may be symptomatic [46]. Priority should be given to improve symptom control. Consequently, clinicians must discuss potential risks and treatment-emergent adverse events in addition to potential benefit from therapy, and early palliative care referral should be considered [46]. In women with a good performance status (PS), participation in clinical trials is highly recommended.

Response rates to single-agent ChT, such as weekly paclitaxel, PLD, topotecan, gemcitabine, or metronomic cyclophosphamide are low, averaging 5–15% and median OS of 12 months [46]. Studies in this setting have been designed until progression or unacceptable toxicity. There is no robust data comparing these regimens and the choice may be guided by toxicity profile and patient preferences. Yet, patients with poor PS should be considered for best supportive care only.

Trabectedin–PLD is an option for patients with TFIp > 6 months unable to receive further platinum-based ChT [47].

The AURELIA (NCT00976911) phase III trial assessed the role of bevacizumab in combination with single-agent ChT (weekly paclitaxel, PLD, or topotecan). The addition of bevacizumab resulted in improved PFS and quality of life, with no statistically significant differences in OS. Nevertheless, the study was not powered to detect these differences and 40% of patients cross-overed to bevacizumab. Weekly paclitaxel and bevacizumab demonstrated better outcomes that the other two arms [48].

Novel therapeutics, including antibody drug conjugates have shown promising results. The MIRASOL (NCT04209855) trial demonstrated that mirvetuximab soravtansine improved PFS and OS in high FRα expression platinum-resistant ovarian cancer, when compared to single agent ChT [49]. Approval from European and Spanish healthcare authorities is pending.

### Recommendations

- Secondary cytoreduction should be contemplated in selected patients [I, A].
- For cases in which platinum is the best option, (1) bevacizumab and a platinum doublet (if no prior Bev) or (2) platinum doublet followed by PARPi maintenance (if response to platinum and no prior PARPi) regardless of BRCA and/or HRD status, can be considered [I, A]. For subjects with a highly symptomatic relapse or requiring a rapid response, combination with bevacizumab is the preferred approach [III, B].
- When platinum is not an option, single-agent non-platinum alternatives can be considered [I, B]. The addition of bevacizumab should be recommended in those without contraindications (IA). Early referral to palliative care should be considered [II, A].

### Follow-up, long-term implications, and survivorship

There is no consensus regarding the optimal follow-up strategy for ovarian cancer survivors. Table 3 can be used as a general guideline. Given that some patients can suffer from late relapses, extended follow-up beyond 5 years can be considered for some patients. For BRCA1/2 mutation carriers, high-risk breast cancer screening guidelines should be followed, although some retrospective studies have reported a low rate of metachronous breast cancer in EOC patients [1]. With the prolonged survivals achieved in the era of PARPi, long-term toxicity, such as long-lasting neuropathy, sexual impairment, or myelodysplastic syndromes associated with ChT and PARPi treatments, should be carefully monitored throughout follow-up (Table 4) [50].

**Table 3:** EOC follow-up recommendations

	0–2 years from end of treatment	2–5 years from the end of treatment
Review of symptoms	Every 3 months	Every 6 months
Physical examination	Every 3 months	Every 6 months
Pelvis examination (gynaecologist)	Every 3 months	Every 6 months
Blood test + Ca125/HE4	Every 3 months	Every 6 months
TC scan	Every 3–6 months	Every 6–12 months or depending symptoms

**Table 4:** Summary of recommendations

Diagnosis and staging	Screening for EOC in average risk women is not recommended	I, E
	The initial evaluation of a patient with suspicion of EOC should include physical examination, laboratory testing with CA-125, and pelvic ultrasonography	II, A
	CT of the thorax, abdomen, and pelvis is recommended in the diagnostic workup to assess the extent of the disease	II, A
	Laparoscopic surgery can be considered in advanced EOC to assess cytoreduction, obtain material for pathologic diagnosis, and predictive biomarkers	II, B
Pathology and molecular biology	Initial IHC-based diagnosis should be performed by a gynaecological pathology expert	IV, A
	All patients with non-mucinous EOC should be tested for germline/somatic BRCA1/2 mutation at primary diagnosis to discard a hereditary syndrome, obtain prognostic information, and select a biomarker of response to PARPi	I, A
	MMR testing and/or more extensive germline NGS panels are recommended according to clinical suspicion and family history of cancer	II, A
	Determination of HRD with a clinically validated test is strongly recommended at initial diagnosis in every high-grade EOC to provide prognostic and predictive biomarker information	I, A
Management of early stage disease	Surgery should establish the extent of the disease by means of an appropriate staging approach, which makes it possible to establish the subsequent indication for systemic treatment	I, A
	Platinum-based ChT is recommended in all cases of high grade or stage II	I, A
	The most widespread option is the combination of carboplatin and paclitaxel, although carboplatin monotherapy may be acceptable	II, A
	The duration of treatment should be at least 3 cycles, although in HGS cancers the recommendation should be 6 cycles	II, A
Management of advanced stage disease	Cytoreductive surgery with the aim of achieving complete cytoreduction (absence of all visible residual disease) is the cornerstone of treatment for advanced EOC and should be performed in specialised centres	II, A
	Lymphadenectomy in primary completely debulked AOC with clinically negative lymph nodes is not recommended	I, A
	Standard treatment is based on a combination of carboplatin (AUC 5–6) and paclitaxel (175 mg/m2) every 3 weeks for 6 cycles	I, A
	If complete cytoreductive surgery is not feasible, NACT for 3 cycles followed by ICS and 3 cycles of ChT is recommended	I, A
	IP ChT and HIPEC are not regarded as standard of care	I, D
	Bevacizumab should be considered in addition to ChT, especially in patients with stage III and residual disease or stage IV	I, A
	Maintenance treatment with PARPi, bevacizumab or both is recommended after ChT in HG-EOC. PARPi can be recommended if partial or complete response or no evidence of disease after first-line platinum-based chemotherapy. Treatment decision should be based on molecular and clinical factors. Recommended alternatives (not in order) are as follows • In BRCA1/2-mutated patients: single agent with olaparib, rucaparib or niraparib, or combination of olaparib plus bevacizumab • HRD tumours: single agent with niraparib, rucaparib or combination with olaparib plus bevacizumab • HRP tumours: niraparib, rucaparib or bevacizumab	I, A I, A I, A
	Paclitaxel–carboplatin ± bevacizumab is the standard systemic chemotherapy used in non-HGSC	I, B
Management of recurrent disease	Secondary cytoreduction should be considered in selected patients	I, A
	For patients in whom platinum is the best option, 1) bevacizumab and a platinum doublet (if no prior bev) or 2) platinum doublet followed by a PARPi maintenance (if response to platinum and no prior PARPi) regardless of BRCA and/or HRD status, can be considered • In cases with a highly symptomatic relapse or requiring a rapid response, the preferred approach is combination with bevacizumab	I, A III, B
	For subjects for whom platinum is not an option, single-agent non-platinum options may be contemplated • The addition of bevacizumab should be recommended in those with no contraindications • Early referral to palliative care should be considered	I, B I, A I, A
Follow-up, long-term implications, and survivorship	Follow-up of EOC survivors should include at the very least, review of symptoms, physical and pelvic examination, and CA-125 until 5 years after the end of treatment	IV, B
	Long-term follow-up beyond 5 years after the end of treatment can be considered in some cases	III, B
	Long-term toxicity should be monitored throughout follow-up	V, C

### Recommendations

- As a minimum, follow-up of EOC survivors includes reviewing symptoms, physical and pelvic examination, and Ca125 until 5 years after the end of treatment [IV, B].
- Long-term follow-up beyond 5 years after the end of treatment should be considered for some patients [III, B].
- Long-term toxicity should be monitored throughout follow-up [V, C].

## Data Availability

Not applicable.
